# VetCompass Australia: A National Big Data Collection System for Veterinary Science

**DOI:** 10.3390/ani7100074

**Published:** 2017-09-26

**Authors:** Paul McGreevy, Peter Thomson, Navneet K. Dhand, David Raubenheimer, Sophie Masters, Caroline S. Mansfield, Timothy Baldwin, Ricardo J. Soares Magalhaes, Jacquie Rand, Peter Hill, Anne Peaston, James Gilkerson, Martin Combs, Shane Raidal, Peter Irwin, Peter Irons, Richard Squires, David Brodbelt, Jeremy Hammond

**Affiliations:** 1 Sydney School of Veterinary Science, Faculty of Science, University of Sydney, Sydney, NSW 2006, Australia; paul.mcgreevy@sydney.edu.au (P.M.); navneet.dhand@sydney.edu.au (N.K.D.); 2 School of Life and Environmental Sciences, Faculty of Science, University of Sydney, Sydney, NSW 2006, Australia; peter.thomson@sydney.edu.au; 3 Charles Perkins Centre and School of Life and Environmental Sciences, University of Sydney, Sydney, NSW 2006, Australia; david.raubenheimer@sydney.edu.au; 4 Faculty of Veterinary and Agricultural Sciences, University of Melbourne, Werribee, VIC 3030, Australia; cmans@unimelb.edu.au; 5 School of Computing and Information Systems, University of Melbourne, Parkville, VIC 3010, Australia; tb@ldwin.net; 6 School of Veterinary Science, University of Queensland, Gatton, QLD 4343, Australia; r.magalhaes@uq.edu.au (R.J.S.M.); j.rand@uq.edu.au (J.R.); 7 Child Health Research Centre, University of Queensland, South Brisbane, QLD 4101, Australia; 8 School of Animal and Veterinary Sciences, University of Adelaide, Roseworthy, SA 5371, Australia; p.hill@adelaide.edu.au (P.H.); anne.peaston@adelaide.edu.au (A.P.); 9 Faculty of Veterinary and Agricultural Sciences, University of Melbourne, Parkville, VIC 3010, Australia; jrgilk@unimelb.edu.au; 10 School of Animal and Veterinary Science, Faculty of Science, Charles Sturt University, Wagga, NSW 2650, Australia; macombs@csu.edu.au (M.C.); shraidal@csu.edu.au (S.R.); 11 School of Veterinary and Life Sciences, Murdoch University, Murdoch, WA 6150, Australia; p.irwin@murdoch.edu.au (P.I.); P.Irons@murdoch.edu.au (P.I.); 12 College of Public Health, Medical and Veterinary Science, James Cook University, Townsville, QLD 4811, Australia; richard.squires@jcu.edu.au; 13 Pathobiology and Population Services, Royal Veterinary College, University of London, Hertfordshire AL9 7TA, UK; dbrodbelt@rvc.ac.uk; 14 Information and Communications Technology, University of Sydney, NSW 2006, Australia; jeremy.hammond@sydney.edu.au

**Keywords:** companion animals, canine, feline, equine, disease surveillance, veterinary, electronic patient record, epidemiology, big data

## Abstract

**Simple Summary:**

The VetCompass Australia program collects real-time clinical records from veterinary practices and aggregates them for researchers to interrogate. It delivers Australian researchers sustainable and cost-effective access to authoritative data from hundreds of veterinary practitioners, across Australia and opens up major international collaborative opportunities with related projects in the United Kingdom and elsewhere.

**Abstract:**

VetCompass Australia is veterinary medical records-based research coordinated with the global VetCompass endeavor to maximize its quality and effectiveness for Australian companion animals (cats, dogs, and horses). Bringing together all seven Australian veterinary schools, it is the first nationwide surveillance system collating clinical records on companion-animal diseases and treatments. VetCompass data service collects and aggregates real-time, clinical records for researchers to interrogate, delivering sustainable and cost-effective access to data from hundreds of veterinary practitioners nationwide. Analysis of these clinical records will reveal geographical and temporal trends in the prevalence of inherited and acquired diseases, identify frequently prescribed treatments, revolutionize clinical auditing, help the veterinary profession to rank research priorities, and assure evidence-based companion-animal curricula in veterinary schools. VetCompass Australia will progress in three phases: (1) roll-out of the VetCompass platform to harvest Australian veterinary clinical record data; (2) development and enrichment of the coding (data-presentation) platform; and (3) creation of a world-first, real-time surveillance interface with natural language processing (NLP) technology. The first of these three phases is described in the current article. Advances in the collection and sharing of records from numerous practices will enable veterinary professionals to deliver a vastly improved level of care for companion animals that will improve their quality of life.

## 1. Background

The analysis of data on clinical management of companion-animal disorders provides critical evidence on the ranking of key conditions affecting animal health and welfare, as well as an evidence base for the cost-effectiveness of competing treatments and care options for these animals. Australia has one of the highest rates of pet ownership in the world, with 38.5% of the 8 million Australian households owning at least one dog and 29.2% owning a cat [1]. There are also approximately 570,000 pleasure horses in Australia [2]. The widespread distribution of companion animals means that their health and welfare have a significant and direct impact across a broad socio-economic cross-section of the Australian population. Many areas of companion-animal research also have direct translations to and implications for human health, such as investigations into zoonoses (e.g., rabies and Hendra virus).

This article describes a collaborative project that builds on a similar approach adopted by the lead author and others at the Royal Veterinary College (RVC), UK. VetCompass Australia uses a software platform established at the RVC to harvest clinical records from veterinary practices into a centralized resource, enabling access to comprehensive and real-time primary practice data. It has been running in the UK for seven years, and is already transforming veterinary science there as the leading source of epidemiological data, with nearly 40 million treatment records related to approximately 6 million animals, from 498 practices available for interrogation [3,4]. In Australia, sentinel surveillance approaches have been used for a number of companion-animal diseases. The Disease WatchDog surveillance system was a prospective national disease-surveillance project, focusing on dogs and cats [5]. Data entered into the system from veterinary clinics as cases of diseases were diagnosed provided monthly online updates. However, recent analysis of its data has indicated the existence of significant data artefacts that are likely to be a result of poor compliance by users of the system [6]. The VetCompass data service overcomes these challenges in that, once implemented on practice-management systems in Australia, it allows automated transmission and de-identification of veterinary clinical records into a centralized database. Researchers can then immediately interrogate the electronic records held by participating clinics. As a long-term investment, the research potential grows as new records are entered and as new practices sign up. In addition, the project will incorporate new linguistic programing methodology to improve the quality of data extraction from those records.

The project will significantly and rapidly advance the understanding of companion-animal diseases in Australia. Bringing together all seven Australian veterinary schools, the project is based on a consortium that operates a shared resource to support the most cost-effective mechanism to undertake companion-animal veterinary research: epidemiology on aggregated electronic patient records. The Australian government has provided support through the Australian Research Council’s (ARC) Linkage Infrastructure and Equipment Fund (LIEF), which will enable the comprehensive reporting of data that are collected routinely in Australian veterinary clinics but are not currently integrated and are so far virtually untapped. This is the single most important development required to support veterinary research across a range of fields, including public health. It is expected to transform veterinary-science research and the curricula at all Australian veterinary faculties.

The project has been designed to develop across three essential phases of work: (1) roll-out of the VetCompass platform to harvest Australian veterinary clinical data; (2) continued development of the data interrogation platform to allow researchers and clinicians to realize their research potential; and (3) creation of a world-first real-time surveillance interface with natural language processing (NLP) technology. The VetCompass platform will open the way for collaborating veterinary schools to prioritize research goals and monitor the emergence and/or re-emergence of biosecurity threats that are unique to Australia as an island nation. The project is now entering Phase 2 and can be contacted via www.vetcompass.com.au.

**Figure 1:**
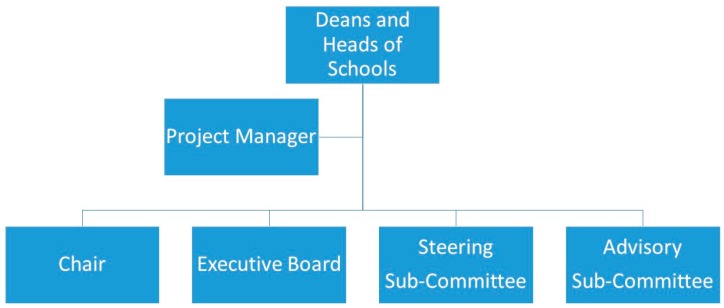
A diagram to illustrate the organizational framework of VetCompass Australia.

As of August 2017, the LIEF Consortium (the 15 co-investigators on the ARC grant, who represent all the Australian veterinary schools) has agreement from 180 Australian veterinary clinics to participate in the VetCompass program, and has commitments from industry and peak body partners to increase this to 80% of Australian veterinary practices. The University of Sydney’s Human Research Ethics Committee has granted approval to harvest historic and future data from episodes of veterinary care (Approval number: 2013/919). The organizational framework appears in Figure 1.

## 2. How It Works

When veterinary clinics express interest in taking part in VetCompass Australia, they are given a copy of the Participant Information Statement, which outlines the project and has HREC approval. They then complete a Practice Consent Form. They have the option to withdraw from the study at any point, but any data already transferred remains for the use of researchers.

VetCompass Australia mirrors its UK sister project. The platform (www.VetCompass.org) has two main components. Firstly, there are web-services with a single page application (angular) user interface for these web-services. Some of the web-services will be hosted by the University of Sydney to service requests originating in Australia, while others are hosted in and federated to the UK to service global requests. Secondly, there are back-end databases that include a Structured Query Language (SQL) server for persistence, disaster recovery, and staging data as they are imported, and a search database for searching and other denormalization of the persisted data.

**Figure 2:**
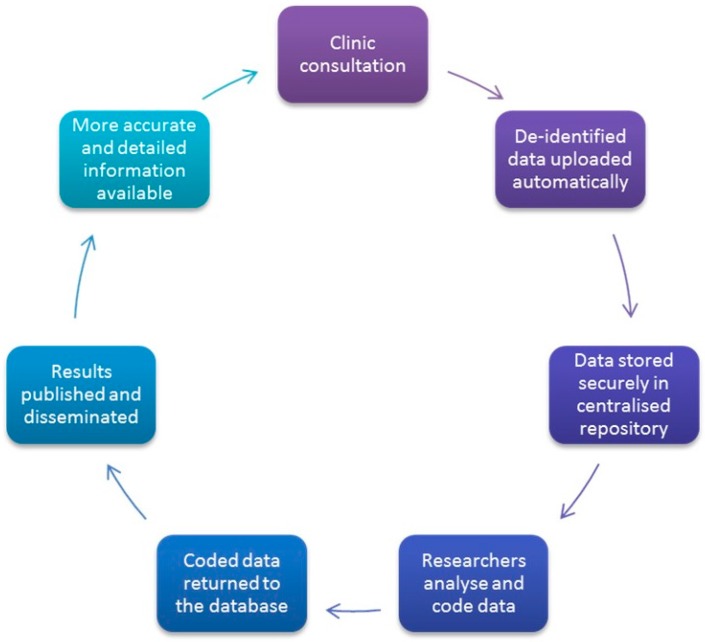
An illustration of the flow of data through the VetCompass Australia system.

VetCompass extracts data directly from practice management software (PMS) so that no extra work is required of clinicians (see Figure 2). The VetCompass operations team, in collaboration with the practice owners, implement an integrated and automated harvester on each clinic site (via application programming interfaces). This harvester is part of the development undertaken by the project. This aligns with our commitment to observe practice without affecting care. Extracted data are uploaded directly from practices’ databases via our web-services platform to the VetCompass database. The uploads vary with practices/groups but are frequently on a daily basis (e.g., overnight).

Data uploaded as described above are pushed via encrypted web-services to the secure VetCompass database where they are imported into the relevant database tables. After the initial import of a dataset, these data are checked for internal inconsistencies, such as breed versus species data, and whether all records appear to have been uploaded, for example, presence of free-text notes. Data are evaluated in detail during research studies within the online interface. 

The data are stored securely in the University of Sydney’s servers. We have undertaken best practice information security procedures, conducting architecture reviews, and independent third-party penetration tests. The consortium owns all the data and other material contained within the database, including any data or other material entered by the researchers using the system. Aspects of the metadata are shared with the Royal Veterinary College to assist in making improvements to the platform.

All aspects of the study are treated as strictly confidential, and only researchers in VetCompass Australia or collaborating research projects, such as VetCompass at the RVC, have access to the raw data. We abide by Australian privacy legislation. Individual practices, animals, or clients are not identifiable in any publications resulting from research using this system. 

Personal details of the owners are removed from the record before reaching researchers. The only exception to this is the postcode, which is collected to allow for geographical analysis of the data for certain studies. The animals themselves are not named; instead, each is given a unique identifier. This allows us to collate complete case histories from separate visits. We also collect the animal’s microchip number so that its record can be traced across different clinics, specialists, and owners. This also allows us to mesh patient data with records from owners who use the smartphone app doglogbook (www.doglogbook.com).

Owners who do not want their animals to be a part of this project can notify clinic staff members, who then mark the record accordingly. We then remove them from data transfers and do not use their records in any studies.

With a focus on extraction and cleaning of data from numerous PMSs, the team at the RVC has developed a canonical form which offers a very simple data schema for sending data. The canonical form is implemented in various formats, such as XML Schema Definition, which allows for the selection of optimal implementation for each PMS. The development team is engaged in ongoing work with either the PMS developers or the in-house developers from practice groups to map model PMS data to the canonical form. This process facilitates the import data from the canonical form into VetCompass database. Once they have been imported, the data are subjected to automated checks for common problems and undergo a validation process. Around 60–70% of practices in Australia use RxWorks. The current software handles the extraction of data from RxWorks and is made available to in-house developers at practice groups to deploy and direct at their own databases. 

The VetCompass Australia facility has its project manager and server based at the University of Sydney. There is commitment from the Australian schools to support the facility for the next four years. It is anticipated that nationally competitive grants and industry linkage will ensure the facility is sustainable in the longer term. The resource will be available to all Australian veterinary and allied researchers at all times through the application to the Executive Board, that meets every month and comprises the project manager and representatives of six of the contributing schools (one school contributes less funding than all of the others and so has agreed to attend only the annual meeting of the Board, in a non-voting capacity). 

It is anticipated that researchers will access the coding portal via a web interface and create a focal dataset (e.g., all records related to cats with a diagnosis of chronic renal disease). They then further code and characterize the data (e.g., by answering questions related to the diagnostic techniques implemented, as evidenced by the free-text clinical records). These secondary data are then automatically saved to the VetCompass Australia database, and, when finished, the researcher can export this data interrogation with related animal and treatment fields to a spreadsheet for subsequent analysis.

Each veterinary school has existing or proposed studies that will interrogate VetCompass data. Some examples of projects already waiting for the resource are: the identification of spatial clustering of clinical cases of zoonotic infections; the development of predictive spatial models of clinical cases of zoonotic infections and identification of areas at most risk of clinical zoonotic infections; epidemiology and lifestyle factors associated with body condition score and diabetes; criteria-based and population-validated diagnostic systems for dermatology treatments; and the use of antimicrobials in the companion-animal sector and development of guidelines that instigate changes in usage to reduce the development of resistant organisms.

There are three main types of enquiry that the VetCompass facility invites: student, strategic, and major research projects. Student projects are intended chiefly for final-year veterinary student research projects and will typically focus on simple enquiries (e.g., breed-specific enquiries). These reports could describe the longevity and the demographics of the breed. For every condition to which a breed is predisposed, the students could report on the temporal and geographic distribution as well as risk factors and treatments. Several students may focus on one breed at the same time, and, for group projects, teams of students may code the same records. Strategic projects are designed for clinical academics, or early to mid-career researchers from the participating institutions. Importantly, strategic projects must not encroach directly on research questions that have been ear-marked for major research projects. The success of applications to VetCompass Australia does not depend on the track-record of the investigators. Instead, these projects are reserved for academic staff other than full professors and are capped at two concurrent projects per chief investigator. 

Any researcher wanting to access the data held in the VetCompass system needs to apply to the Board, which uses set criteria to determine eligibility. The process followed depends on the type of project proposed. Student projects are allocated according to the local rules for each university, with the Board members from each institution selecting breeds in advance. Strategic project submissions are sought from each university then brought to the Board for ratification. Where the topic relates directly to a current major project, the Chief Investigator on the relevant major project must also give approval. Ideas for major projects are submitted to the Board for review, who must agree on whether or not to advance a project. Such approval also depends on current commitments of the data to other projects. 

There will be two calls for applications per year. Multi-year projects will be considered, but priority will be given to shorter projects with a defined outcome and likely output. Major research projects are expected to be led by senior academics within the consortium and with strong track records, since they are intended to be competitive for significant industry collaboration and national funding schemes. With a focus on quality over equity, these projects are largely dependent on the involvement of an epidemiologist as a key member of the investigatory team. Multi-institutional projects are preferred, but not at the compromise of quality.

## 3. Aims and Significance

The need for a nation-wide companion-animal disease-surveillance system to inform rational approaches to companion animal disease control was first triggered by a perceived rise in inherited disorders among companion animals [7,8,9]. Some of these are related to breed standards [10], while others are not [11]. Recent large-scale genomic investigations have clearly identified increased deleterious genetic variation in domestic dogs compared with ancestral canids, extending earlier estimations of multiple deleterious gene carriage [12]. VetCompass UK was developed as part of a response to the need for a surveillance system to describe and evaluate companion-animal disorders, and to formulate plans to eliminate them. Researchers have used the accumulated data related to nearly 6 million animals to publish articles that provide invaluable guidance to clinicians and breeders on breed predispositions and are informing the curricula of veterinary schools globally [13].

Epidemiological studies are powerful means of establishing correlations amongst different types of physical, spatial and temporal data to better understand many aspects of disease. For example, the use of routinely collected data by clinics or government departments facilitates an understanding of how patterns of infectious disease emerge. Outbreak data collected by state governments in Australia aided understanding of the 2007 equine influenza outbreak by allowing estimation of the relative contributions of network and spatial spread, results that will guide planning for control of future outbreaks [14,15]. There are numerous examples of clinical data being used to obtain insights into epidemiology and risk factors for small-animal diseases [16,17,18,19,20]. However, data from specialized clinics at veterinary schools are often used for such analyses where the case population is generally not representative of the general population for which inferences are required, thus resulting in selection bias. In contrast, VetCompass data are aggregated from primary care veterinary clinics, where the case population and case types better represent the general pet population, and therefore, research findings from such data have a greater internal and external validity.

Effective data linkage of clinical records for the development of spatial epidemiological tools is needed to quantify the geographical limits of infectious disease occurrence, the populations at risk, and to identify major risk factors for zoonotic infections of significance to public health. VetCompass Australia will provide the evidence to allow refinement of existing clinical protocols with the aim of implementing targeted control and mitigation strategies to directly decrease animal disease burden.

Companion-animal research is under-funded in Australia, with the chief sources of funding being confined to small non-profit organizations, pet-food manufacturers, and pharmaceutical manufacturers who, quite understandably, have partisan research needs. This can impede long-term, large-scale studies into the burden of disease, risk factors, and cost-effectiveness of treatments for companion animals. The merit of individual therapeutic agents may differ with chronicity and even breed, so it seems clearly beneficial to collect “big” data that may allow clinical guidelines for any disorders of companion animals to be fine-tuned as more evidence becomes available. Another good example concerns companion-animal vaccinations. VetCompass will enable researchers to investigate the extent to which Australian veterinarians follow international vaccination guidelines [21] and improve surveillance and understanding of vaccine-related adverse reactions, which are thought to be under-reported.

Therapeutic guidelines are needed more than ever as the veterinary profession takes a leadership role in monitoring the use and abuse of antimicrobial agents that lead to antimicrobial resistance [22,23]. Microbial pathogens of companion animals cause disease in owners and allow antimicrobial resistance to emerge in humans. Methicillin-resistant *Staphylococcus aureus* (MRSA) is a recognized problem in equine hospitals and a recent study found that equine veterinarians had a very high rate of MRSA carriage [24,25]. MRSA has been transmitted between humans and domestic pet dogs and cats in both directions—zoonotic and reverse-zoonotic transmission [26]. MRSA costs Australian taxpayers between $70 million and $300 million in preventable human infections, and VetCompass will advance our understanding of the appropriateness of veterinary antimicrobial usage and the potential role of poor antimicrobial stewardship on resistance transference between pets and their owners.

The potential and power of natural language processing (NLP) to improve analysis of large or poorly coded datasets is considerable, and within the veterinary context this technology offers some particularly exciting possibilities when compared with human health initiatives. Existing health applications have tended to focus on either very small-scale datasets (e.g., hundreds of patients [27]) or publicly available data sources such as Twitter, with the incumbent issues of data provenance, and imprecise location data [28,29]. Emergent work on the application of NLP in the analysis of clinical notes for human patients and monitoring of disease outbreaks [30,31,32] is severely limited because patient privacy legislation limits access to these free-form notes. In contrast, access to high-quality de-identified free-form clinical notes within data in VetCompass will be relatively unfettered.

## 4. The Relevance of VetCompass to the Needs of Companion-Animal Health

The paucity of comprehensive epidemiological data of clinical relevance is a problem facing almost all veterinary researchers in Australia, and the cost for any individual institution to address this gap is prohibitive. Recruiting veterinary clinics and their personnel to assist with active disease surveillance on a per-project basis is time-consuming and inefficient: it runs the risk of “respondent fatigue” among practitioners and progressive disinterest in data collection. The consortium has already had expressions of interest from more than 50 researchers at Australian universities seeking the types of data collected by VetCompass.

The project will support a wide range of research disciplines in key species and diverse breeds. There are enormous positive implications for the welfare of Australia’s companion animals and, ultimately, any animal for which individual records are kept, by providing the opportunity to evaluate the lifetime impact of disorders on the welfare of affected animals [33,34]. The prospects for longitudinal studies of individual animals are particularly exciting. Financial and policy gains for the public, industry, and government agencies will be made through research outcomes that promote cost-effective treatments, create better breeding guidelines, and document biosecurity risks.

The VetCompass Australia project will support a wide range of existing research projects as it provides both baseline data that can be augmented and an internally validated source of information. This is one possible means of ranking disorders and prioritizing prevention, eradication and management schemes, and research plans [35]. Prioritization can help funding bodies, such as the ARC and the Australian Government’s Department of Agriculture, to identify research priorities and effectively assess the value of investment in particular ventures. It will also assist the cat, dog, and horse breeders of Australia to decide how to prioritize inherited disorder disease mitigation programs and monitor the progress of these initiatives over time. This level of analysis is not currently possible and, at a national scale, could provide a significant return on investment.

## 5. Relevance of VetCompass to the Needs of Veterinary Clinics

Evidence-based practice is the science of making informed, validated decisions for day-to-day clinical practice scenarios. This ideal is currently difficult to implement in the veterinary practice context because of the limited availability of high-quality data. Unfortunately, some clinical decisions in veterinary practice are, of necessity, based on evidence generated in case studies, personal experience, extrapolations from human medicine, or advice from colleagues, which is considered the lowest level of evidence [36,37]. VetCompass will allow researchers in companion-animal medicine and surgery, as well as in the para-clinical sciences such as pathology, microbiology, virology, and parasitology, to benefit from the collective clinical experience of their colleagues in a national network of private practices and at other participating institutions. In aggregating these data, the Australian veterinary profession will, for the first time, generate a sound, broad-based dataset from which to make evidence-based recommendations for best practice in the diagnosis, treatment, and prevention of companion-animal diseases.

In Australia, there is almost no published information on age-, sex- and breed-based disorder prevalence and treatment for dogs. Equivalent data on cats and horses are even more limited. VetCompass offers the first opportunity in Australia to gather baseline demographic statistics (such as breed, age, sex, neuter status, insurance status, and bodyweight) on Australian dogs, cats, and horses. It will generate estimates of disorder prevalence for the most common disorders in the most common breeds and crossbreeds. This will identify risk factors for common disorders and reveal the most cost-effective treatments and their impact on animal survival and health. In addition, the data derived from this project may inform strategic breeding programs to improve the health and longevity of vulnerable breeds.

## 6. Use of VetCompass Data to Understand Patterns of Zoonotic and Emerging Infectious Diseases for Gaining Insight for Both Human and Animal Health

It is anticipated that VetCompass data will become the primary source of information about temporal and spatial patterns in distribution of zoonotic and emerging infectious diseases in companion animals in Australia. This will not only enable early detection of diseases but also allow health authorities to monitor changes in disease incidence that will inform policies for their control. This will have positive implications for the health and wellbeing of both companion animals and humans.

Emerging infectious diseases in humans and wildlife populations are increasing worldwide, alongside other forms of global change [38,39,40]. A major challenge for science is to understand pathogens in terms of how they emerge, lie dormant, and sometimes disappear, and of how they drive aspects of population immunity. For conservation, production, and human health, there is an imperative to attain better data on the factors that influence the movement and transmission of disease from wildlife to humans. This affects, in part at least, the push for a One Health approach to the understanding of zoonoses [41,42,43].

VetCompass will provide a highly efficient and economical, passive and non-invasive system for monitoring and surveillance of companion-animal diseases of public health importance. Practice records will be uploaded daily automatically to the centralized repository. The real-time nature of the data means that information about the temporal and spatial distribution of diseases will be available as diseases occur. With disease modeling and further automation, VetCompass data will be useful for public health authorities and veterinary bodies by providing information on trends in disease occurrence, and predicting where and when diseases are likely to occur.

Through real-time surveillance of incoming data, the VetCompass platform could be instrumented to communicate alerts of emergent diseases, such as rabies or Hendra virus (e.g., if a cluster of veterinary practices starts to record neurological signs in multiple patients). The project also has the capacity to reveal how wildlife health may integrate with domestic animal and human health. Many veterinary clinics throughout Australia also attend to a large number of sick and injured wild animals during the course of their normal work, but most of the information that could be acquired from such accessions is not currently captured or analyzed by Wildlife Health Australia (WHA), which is the key group responsible for wildlife health surveillance in Australia (www.wildlifehealth.com.au). WHA currently uses sentinel clinic surveillance to capture such data and could clearly benefit from wider sources, since summary reports on a selection of wildlife disease and mortality events are published quarterly through the National Animal Health Information System (NAHIS) in each issue of Animal Health Surveillance Quarterly (AHSQ). WHA also provides information to help fulfil Australia’s reporting requirements to the World Organization for Animal Health (OIE). One caveat with wildlife data is that, in the absence of unique identifiers, such as microchips, one animal could theoretically be presented with the same disorder several times. Therefore, the prevalence data that emerge from wildlife would need to be interpreted with caution. Additionally, many practices do not create formal medical records for presented wildlife.

## 7. Use of VetCompass Data for Training Future Veterinary and Animal Scientists, and for Guiding Development of Veterinary Curricula

VetCompass data will be invaluable for training future veterinarians and animal scientists in epidemiology, biostatistics, clinical research and evidence-based practice. Most veterinary schools require veterinary and animal science students to undertake courses in research and enquiry, and to conduct research projects. For these students, as well as for full-time Honours or PhD research students, a national data-collection and mining platform offers a dynamic, high-quality and pre-funded dataset for self-contained research projects or modules of larger projects. The VetCompass project also has potential for facilitating the development of research careers of clinical academics with a substantial clinical service workload that reduces the time they have available to conduct primary research.

Much of the data harvested by VetCompass from electronic clinical records will be in plain text, providing myriad opportunities for NLP. Applications of NLP to clinical data have historically been marred by data-access restrictions and sparsity, but the volume of data collected by VetCompass is conducive to the application of pre-training of computational models, which has been shown to substantially boost accuracy [44,45]. There is significant value in the innovative NLP components of VetCompass. The consortium includes NLP scholars, whose work will boost the functionality of VetCompass by providing researchers with better search and coding tools, and improving clinicians’ coding practices. This is a unique value proposition of the consortium in that research, and the infrastructure enabling that research, deliver mutual benefits. 

The NLP components of the project span a broad spectrum of complexity. For example, among our plans to use NLP in the study of antimicrobial use, a central task will be the determination of the dosage instructions provided to the patient for a given prescription (e.g., “3 × 500 mg tablets daily”), as well as the classification of the diagnosis described in a given clinical note, e.g., via VeNom codes (an online database dictionary maintained by the Royal Veterinary College, see www.venomcoding.org). Methodologically, this is a relatively straightforward classification task but will rely on the availability of hand-coded VeNom codes from VetCompass UK and other sources to train supervised models, and almost certainly will involve extra data annotation. This will be critical in interpreting the context in which antimicrobials have been prescribed (or not prescribed), to aid in the collation of data on the use of antimicrobials in different clinical contexts. The NLP will go hand in hand with machine learning, in capturing and representing uncertainty in the dosage or diagnostic data, and aggregating these imprecise data at the level of species, breeds, clinicians, and practices.

The data-linkage opportunities in this project will also enable innovative research into the medical geography of veterinary diseases in Australia. This includes, for example, linkage with existing projects on the epidemiology of grass-seed foreign-body disease in dogs [46], and projects under development such as the University of Queensland’s Spatial Epidemiology Laboratory (www.spatialepilab.com), the University of Melbourne’s temporo-spatial mapping of cat and dog snake envenomations (SnakeMap) and the University of Sydney’s dog-care and management tracking tool, doglogbook (www.doglogbook.com). The measurable impact of this research will go beyond traditional research outputs. The VetCompass project will allow us to disseminate data and summary information at minimal or no charge to key stakeholders, including veterinarians, breeders, and potential pet purchasers. It is anticipated that feedback loops will permit the tailoring of breed-, sex- and age-specific data so that differential diagnoses will be redefined according to probabilities in the light of these variables. Dissemination of this data to veterinary clinicians will allow for more individualized preventative health programs to be implemented.

In view of the anticipated benefits listed above, it is not surprising that VetCompass has the support of the Heads of all seven veterinary schools in Australia, and support from all the veterinary teaching hospitals based at universities, as they identify a genuine need for the advancement of research in their respective schools and clinics. Participating institutions have agreed to fund an on-going support position for a minimum of four years beyond the ARC infrastructure grant, so that researchers can maximize their interaction with the data over a long period. They are taking joint responsibility for funding, publication, and research output, in that each major contributor is given a seat on the executive board to participate in all governance issues.

The consortium is a comprehensive national group and consists of at least one chief investigator representative from each veterinary school in Australia, and is endorsed by Veterinary Schools of Australia and New Zealand (VSANZ), which was formed to promote strategic research alliances at the highest level of veterinary education. As with the pan-institutional group of veterinary hospital directors, VetCompass is one of a variety of initiatives supported by VSANZ to enhance collaboration across the sector.

The consortium has strong engagement (including co-appointments) with peak bodies such as the Australian Veterinary Association (AVA), the Australian and New Zealand College of Veterinary Scientists (ANZCVS), the Royal Veterinary College (RVC), the UK’s Royal College of Veterinary Surgeons (RCVS), and the Royal Society for the Prevention of Cruelty to Animals (RSPCA) in both Australia and the UK. These representative bodies have given support to VetCompass both financially and in kind.

This project also brings unprecedented opportunities for sharing specialist expertise across institutions and increasing coverage of inter-disciplinary research across the sector. Negotiations that led to formation of the VetCompass consortium focused on equitable access to the resultant data and produced a governance structure (see below, 9. Project Governance) that ensures transparency and accountability.

## 8. Limitations and Challenges

The almost unparalleled scope and size of the dataset notwithstanding, the limitations imposed by the data collection method need careful consideration. Chief among these is the selection bias introduced by the collection of secondary data from animals presented at veterinary clinics rather than from the primary pet population. The availability and utilization of veterinary services is highly variable, as evidenced by the only moderate correlation between the spatial distribution of companion animals and veterinarians in Australia [47]. Spatial effects can be countered to some extent by continuing to broaden the base of data contributors in under-represented areas. However, this selection bias is of a much lower level than that in studies based on data from specialist clinics in veterinary schools or from selection of a convenience sample from the primary pet population. In addition, the data will be collected from a variety of veterinary practices, including rural, urban, and emergency clinics. Inclusion of a variety of clinic types and locations will help overcome the demographic and other inherent biases.

The other major considerations are those inherent to multi-source retrospective datasets, mainly arising from an inability to standardize the quality of data collected. The lack of uniformity in the diagnostic criteria, clinical management, and record-keeping across the large number of clinicians could result in misclassification and confounding biases. This conundrum is termed data-rich, information-poor (DRIP), but actions can be taken to ensure that the data can be mined appropriately [48]. Such measures include the application of robust scientific methods during the data cleansing, analysis, and interpretation phases. Additionally, there is some evidence that functional data-testing can overcome bad data or data-quality issues [49].

Another challenge is the limited skill of veterinary students, and veterinarians in general, to handle, manage, and analyze data, particularly big data generated by surveillance systems such as VetCompass. The primary focus of veterinary education is to train students in the treatment, prevention, and control of animal diseases, but there is limited focus on providing training in data management and analysis. Many other disciplines, such as business studies, have increased the emphasis on data analytics in response to big data from social media and businesses. It is anticipated that veterinary schools will need to keep pace and increase emphasis on training in data-handling and analysis in the veterinary curriculum, or offer specialized courses to interested students and veterinarians.

## 9. Project Governance

The shared facility is governed by an Executive Board, a steering sub-committee, and an advisory sub-committee. The Executive Board consists of senior representatives from six of the major contributing veterinary schools (one school elected to make a relatively minor financial contribution and accordingly agreed to step away from Board responsibilities from the outset). The Board holds monthly meetings to plan and review the VetCompass build-and-deployment progress. It is also responsible for reaching consensus on financial matters, personnel hiring, data access, citation and acknowledgment requirements. From time to time, the Board delegates tasks and responsibilities to selected groups from the Steering and Advisory committees for considering critical matters of eResearch infrastructure, data management, and deduplication of research topics. The ongoing nature of the VetCompass project means that, over time, participating veterinary schools may need to replace their nominated Board representative with another chief investigator. During the rollout and implementation of Phase 1, the steering sub-committee (comprising the ARC grant holders, the project manager, software engineers, and up to 10 volunteers invited by the Board from the Australian research community) reviews and advises on functionality and use of VetCompass. The non-executive advisory sub-committee advises the Board on areas of expertise, and provides ongoing outreach through publication, conference papers, social media, research supervision, and scholarly user support for VetCompass. It consists of Board-appointed project collaborators and specialist advisors to advise the Board on matters relating to the building and deployment of VetCompass. It includes an NLP specialist and representatives of the RVC, the RSPCA Australia, and the AVA. Additional members may be appointed at the Board’s discretion.

The VetCompass platform comprises two main data systems: the Backend Database and the Analysis Portal. With the provided LIEF funding, both systems are being developed to maximize access for Australian researchers. The portal is available 24/7 and will support concurrent access. The backend database collects data records from participating veterinary clinics and stores them in a hosted SQL database with password-protected access. Access to this database is restricted to the collection manager (the Project Manager of the LIEF grant) and Executive Board members. The system is currently designed to host 200 million animal records, which is estimated to be sufficient until 2020. Federation of the backend database with the UK and other international data is a key outcome of the current government funding. De-identified and aggregated data will be available to Australian researchers through the analysis portal, designed to facilitate research projects and provide searching, aggregation, downloading, and coding services. Access to VetCompass requires authentication of approved researchers. Further improvement to the existing portal is a core aspect of the project.

VetCompass in the UK has been highly productive with the publication of peer-reviewed mortality studies on dogs [3] and cats [4]; longevity and prevalence reports on several breeds (e.g., Cavalier King Charles spaniels [50], pugs [51] and German Shepherd dogs [52]); multi-breed assessments of gastric dilation-volvulus [53], dystocia [54], diabetes mellitus [55], and audits of anti-microbial usage in companion animal practice [56]. The Australian consortium plans to apply the same methods to data on local dogs and publish matching reports so that comparison between breeds in the two countries can be made.

It is anticipated that VetCompass in Australia will continue to grow in unison with its sister project in the UK and that, together, a family of similar projects using the same approach to electronic veterinary patient records will be established throughout the developed world.

Access is mediated by a chief investigator of a given researcher’s institution and the Project Manager. These gatekeepers will also actively monitor proposed research questions to minimize the duplication of research. The data-collection process includes de-identification to comply with Australian privacy laws.

## 10. Conclusions

VetCompass Australia has brought together all seven of the veterinary schools in Australia and is the first Australia-wide surveillance system that collects real-time records from veterinary clinics and aggregates them for researchers to monitor and record companion-animal diseases and their treatments. It delivers to Australian researchers sustainable and cost-effective access to authoritative data from hundreds of practitioners. These clinical records will reveal longitudinal trends in the prevalence of inherited and acquired diseases and their optimal treatments, revolutionise veterinary clinical auditing in Australia, help the veterinary profession to rank research priorities, and assure research-led curricula in veterinary schools.
